# Facial model collection for medical augmented reality in oncologic cranio-maxillofacial surgery

**DOI:** 10.1038/s41597-019-0327-8

**Published:** 2019-12-09

**Authors:** Christina Gsaxner, Jürgen Wallner, Xiaojun Chen, Wolfgang Zemann, Jan Egger

**Affiliations:** 1 Department of Oral and Maxillofacial Surgery, Medical University of Graz, Auenbruggerplatz 6/1, 8036 Graz, Austria; 2 Computer Algorithms for Medicine Laboratory, Graz, Austria; 3 Institute for Computer Graphics and Vision, Graz University of Technology, Inffeldgasse 16c/II, 8010 Graz, Austria; 4 Shanghai Jiao Tong University, School of Mechanical Engineering, 800 Dong Chuan Road, Shanghai, 200240 China

**Keywords:** Positron-emission tomography, Oral anatomy, Head and neck cancer, Computed tomography

## Abstract

Medical augmented reality (AR) is an increasingly important topic in many medical fields. AR enables x-ray vision to see through real world objects. In medicine, this offers pre-, intra- or post-interventional visualization of “hidden” structures. In contrast to a classical monitor view, AR applications provide visualization not only *on* but also *in relation* to the patient. However, research and development of medical AR applications is challenging, because of unique patient-specific anatomies and pathologies. Working with several patients during the development for weeks or even months is not feasible. One alternative are commercial patient phantoms, which are very expensive. Hence, this data set provides a unique collection of head and neck cancer patient PET-CT scans with corresponding 3D models, provided as stereolitography (STL) files. The 3D models are optimized for effective 3D printing at low cost. This data can be used in the development and evaluation of AR applications for head and neck surgery.

## Abstract (summary)

**Table:** 

Measurement(s)	face • 3D facial model • head and neck squamous cell carcinoma
Technology Type(s)	Positron Emission Tomography and Computed Tomography Scan • computational modeling technique
Sample Characteristic - Organism	Homo sapiens

Machine-accessible metadata file describing the reported data: 10.6084/m9.figshare.11284757

## Background & Summary

During the last ten years, head and neck tumors are increasing in males and females. Since these tumors expand fast and can be disastrous for the patient, an adequate oncologic therapy has to be administered in a short time. In the oro-pharyngeal region, where squamous cell carcinomas (SCC) are the main tumor entity, surgery is the treatment of choice for direct tumor removal. For tumor assessment, CT, MRI or PET scans are widely used in the staging, diagnosis and treatment planning processes, as well as for follow-up controls in clinical centers of oncologic head and neck tumor surgery^1,2^. Especially combined positron emission tomography/computed tomography (PET/CT) scanning through image fusion offers many advantages for the clinical routine in both the tumor staging process and in the oncologic follow-up^3^. These advantages are mainly related to the morphological, CT-based visualization of the anatomical pathology localization, combined with the metabolic PET-based visualization of the pathological radiotracer up-take, which provides a unique fusion of two image modalities. Therefore, one single PET/CT body scan can replace additional radiological investigations^4^. However, since PET/CT images are routinely visualized two-dimensionally on a workstation separately from the patient, a radiological or surgical expert has to mentally map the information gained from 2D imaging to the 3D anatomy of the patient.

Medical augmented reality (AR) aims to eliminate this task by directly enabling visualization of medical data and the patient within the same physical space^5^, basically enabling x-ray vision to see through real world objects. In particular, immersive AR systems, such as optical see-through head-mounted displays (OST-HMDs), can be integrated in the visualization process and can be used to display medical data, in 2D or 3D,directly on the patient without using a separate workstation. A surgeon can look through AR glasses at a patient, while directly examining radiological information about a pathology and the surrounding anatomical structures *in situ*, without looking away from the patient. In oral and cranio-maxillofacial surgery, this new approach for anatomical visualization can support the whole clinical diagnostic and oncological treatment process, by enabling an increased spatial perception of anatomical structures and natural 3D interaction. This is especially relevant in the three-dimensional visualization of head and neck tumors, regarding their anatomical localization or in the visualization of surgical treatment pathways regarding expansive oncologic bone resections. The large interest in this new technology from the community in oral and cranio-maxillofacial surgery has been been demonstrated by several recent studies^6–9^. The key component in a medical AR system is the registration of patient-specific image data to the patient with high accuracy, thus establishing a relationship between physical and virtual space. This can be done in several ways, in example by manually aligning virtual data with the patient^10^ or by using external tracking devices and markers rigidly attached to the patient^11^. However, fully automated image-to-patient registration is most beneficial, as it eliminates the need for complicated calibration and adjustment procedures. Therefore, it is an active field of research^12^.

For the testing and evaluation of such image-to-patient registration algorithms, most research groups resort to commercial standard patient phantoms (e.g. by Sawbones, Vashon Island, WA, USA), which are very cost intensive. Another alternative is the use of plastic dummy heads^13^, however, corresponding medical data for such dummies is not available, and scanning of these models does not result in meaningful medical data, which is needed for more elaborate AR applications, such as different means of visualization or interactive 3D surgery planning, just to name a view. Working with real patients for development and evaluation purposes of such AR systems on the other hand is not feasible, since this process usually takes months, would limit the ongoing clinical patient treatment progress and would be very cumbersome for the already troubled patient. Therefore, the data library presented in this contribution offers a unique collection of 18 F 2-fluorodeoxyglucose (F18-FDG) PET/CT scans of malignant head and neck tumors, as well as stereolithography (STL) files of the patient head. These STL models are optimized for effective 3D printing, enabling users to print their own 3D patient phantoms of the head area at a very low cost. These phantoms can be used for comparative and functional accuracy analyses of image-to-patient registration algorithms in AR. Furthermore, the corresponding PET/CT data provided with this collection can be used to investigate different methods of visualization in medical AR, such as efficient transfer functions^14^ or focus and context volume rendering^15^.

## Methods

The procedures of this study were approved by the ethics committee of the Medical University of Graz, Austria (EK-30-340 ex 17/18, 31-416 ex 18/19, Medical University of Graz, Austria). The trial was registered at German Clinical Trials Register and World Health Organization (registration number DRKS00014853). Informed consent was obtained from all study participants.

**Fig. 1:**
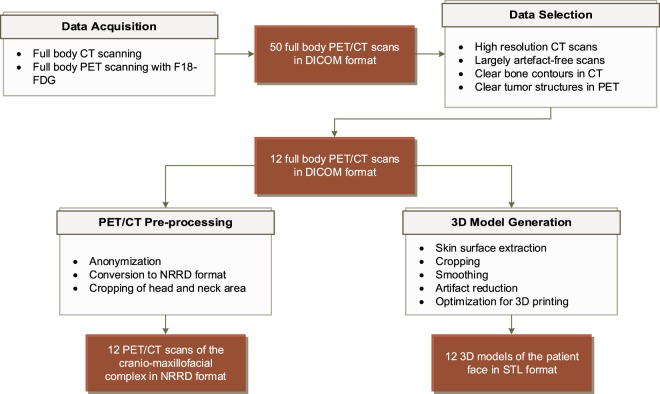
Overview of the workflow to produce the data within this collection. In the data acquisition step, 50 full body PET/CT scans were collected in DICOM format. These scans underwent a thorough screening and selection process, resulting in 12 patient data sets for further processing. On the one hand, these DICOMs were anonymized, cropped and converted to NRRD format for easy further processing. On the other hand, 3D models of the patient’s faces, optimized for 3D printing, were extracted from these scans.

Figure 1 shows an overview of all steps involved in producing the data within this collection, as well as their intermediate in- and outputs. After acquiring 50 full body PET/CT DICOM scans from clinical routine, 12 patient data sets were selected in a thorough screening and selection process. These scans were fully anonymized, converted to Nearly Raw Raster Data (NRRD) format, and cropped in axial direction to enclose the cranio-maxillofacial complex of patients. In a parallel step, the skin surface was extracted from the DICOM data, converted to STL format and optimized for easy, cost-efficient 3D printing.

### Data acquisition and data selection

50 original full body PET/CT scans were retrospectively collected from already existing data sets obtained from the clinical routine in the Department of Oral and Maxillofacial Surgery at the Medical University of Graz, Austria and were provided as DICOM files. Each of the data sets was acquired by performing both a CT and PET scan of a patient with a malignant tumor in the head and neck area. This procedure was done for every case equally according to the clinical standardized cranio-maxillofacial protocol for head and neck tumors. The scans provided within this contribution were performed with a high resolution multi-slice General Electric (GE) Discovery MI PET/CT and/or a Siemens Biograph Vision PET/CT system using a standardized scanning protocol and a B30f reconstruction filter. For all PET/CT scans, F18-FDG was used as a radiotracer. All data sets were acquired within a 3-year period (between 2017 and 2019).

Only high-resolution CT data with an in-plane resolution of 0.98 × 0.98 mm^2^ and a slice thicknesses not exceeding 3.27 mm, as well as corresponding PET scans with clear tracer uptake in the head and neck, a minimal in-plane resolution of 5.47 × 5.47 mm^2^ and minimal slice thickness of 3.27 mm were included in the selection process. Furthermore, no distinction was made between atrophic and non-atrophic facial bones and the tumor location, as these conditions occur patient-specifically. However, incomplete data sets, data sets containing faulty slices or facial structure altering artifacts, as well as data sets including osteosynthesis materials in the facial bones, were excluded from this trial. To ensure the creation of a high-quality and homogeneous collection, only complete data sets of the cranio-maxillofacial area with clear bone contours and clear anatomical tumor structures, without artifacts, were used. According to the inclusion criteria, 12 PET/CT image fusion data sets from the cranio-maxillofacial complex were selected and the remaining data sets had to be excluded for aforementioned reasons. These 12 candidates (n = 12; 9 male, 3 female, 62.5 ± 9.6 years of age) were further processed to form the PET/CT image collection, including patient-specific 3D STL models, for AR system and application testing.

### CT and PET pre-processing

All selected data sets were completely anonymized, de-identified and standardized by converting them from DICOM files into single NRRD files. NRRD files are designed for scientific visualization and image processing of multidimensional data and are therefore especially useful for medical volumetric image data. NRRD files don’t contain patient metadata commonly saved in DICOM tags, like name, patient ID, or birth date. However, the files allow the storage of information such as dimensionality, space units and space origin, which easily facilitates image pre-processing tasks such as cropping, quantization and re-sampling. DICOM to NRRD conversion was performed using the open source software platform 3D Slicer (https://www.slicer.org/). In a second processing step, the anonymized NRRD files were cropped in axial direction to only include the head and neck area of patients. Since the scan resolution between patients, as well as between CT and PET scans is not uniform (see Table 1), the approximate number of slices covering the head and neck region *N* was calculated for each data set individually from the approximate height of the human head *h* and the slice thickness *t* as

1 $$N=\frac{h}{t},$$

where *h* was estimated as 250 mm. The cropped data sets were stored as NRRD files, furthermore, they were converted back to anonymized, standardized DICOMs using 3D Slicer. Both file types are available on figshare^16^.

**Table 1:** Description of data sets included in this collection. For both CT and PET scans, we report on volumetric data set size in voxels and scan resolution in mm^3^. For the 3D model, the total volume size in cm^3^ was determined.

Patient	CT		18F-FDG PET		3D model
	Size	Resolution	Size	Resolution	Size
	(voxels)	(mm^3^)	(voxels)	(mm^3^)	(cm^3^)
1	512 × 512 × 125	0.98 × 0.98 × 2.00	256 × 256 × 83	3.18 × 3.18 × 3.00	18.2 × 16.2 × 16.6
2	512 × 512 × 76	0.98 × 0.98 × 3.27	128 × 128 × 76	5.47 × 5.47 × 3.27	20.0 × 19.3 × 14.1
3	512 × 512 × 76	0.98 × 0.98 × 3.27	128 × 128 × 76	5.47 × 5.47 × 3.27	17.5 × 13.9 × 14.9
4	512 × 512 × 83	0.98 × 0.98 × 3.00	256 × 256 × 83	3.18 × 3.18 × 3.00	18.6 × 14.3 × 14.2
5	512 × 512 × 76	0.98 × 0.98 × 3.27	128 × 128 × 76	5.47 × 5.47 × 3.27	16.5 × 14.3 × 12.6
6	512 × 512 × 76	0.98 × 0.98 × 3.27	128 × 128 × 76	5.47 × 5.47 × 3.27	17.8 × 18.3 × 15.8
7	512 × 512 × 76	0.98 × 0.98 × 3.27	128 × 128 × 76	5.47 × 5.47 × 3.27	16.2 × 13.6 × 14.2
8	512 × 512 × 83	0.98 × 0.98 × 3.00	256 × 256 × 83	3.18 × 3.18 × 3.00	17.9 × 15.0 × 15.4
9	512 × 512 × 125	0.98 × 0.98 × 2.00	256 × 256 × 83	3.18 × 3.18 × 3.00	17.8 × 16.0 × 14.5
10	512 × 512 × 76	0.98 × 0.98 × 3.27	128 × 128 × 76	5.47 × 5.47 × 3.27	17.5 × 14.9 × 14.2
11	512 × 512 × 199	0.98 × 0.98 × 1.25	128 × 128 × 76	5.47 × 5.47 × 3.27	18.3 × 15.9 × 15.6
12	512 × 512 × 76	0.98 × 0.98 × 3.27	128 × 128 × 76	5.47 × 5.47 × 3.27	18.8 × 14.8 × 15.3

### 3D model generation

To generate 3D printable STL files of the facial structure from volumetric CT patient data, the first step is to segment the skin surface of patients from medical imaging and reconstruct a 3D model from it^17,18^. To this end, the selected DICOM files were processed using the free software InVesalius (https://invesalius.github.io/about.html)^19^. InVesalius main features are DICOM image analysis, volume rendering and exporting of files to 3D volumetric formats such as STL. To this end, a CT scan in DICOM format was imported into InVesalius. To define a region of interest for 3D model extraction, the skin surface was segmented from the CT using a pre-defined mask. From this mask, a 3D surface is automatically extracted by the software, which was then exported as STL file for further processing.

**Fig. 2:**
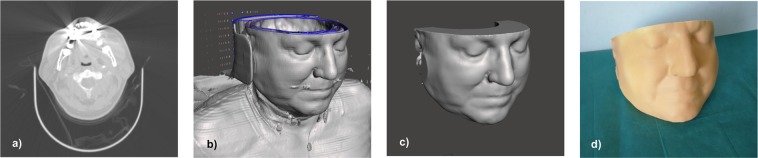
Examples taken from different data processing steps. Figure (**a**) shows an axial CT slice from the lower jawbone region taken from the one of the CT data sets. In (**b**), the skin surface, as reconstructed by the InVesalius software^19^, is shown. Figure (**c**) shows the generated 3D model, fitted for 3D printing, after manual post-processing. Finally, an exemplary 3D print is shown in figure (**d**). Informed consent for publishing these identifiable data was obtained from the participant.

STL files created by the steps described above are not yet suitable for 3D printing. Surfaces extracted from medical image data are usually afflicted by a certain degree of noise. Another difficulty, especially in CT scans from the head and neck area, are dental artifacts originating from metal dental material: dental hardware produces distinct streaking artifacts which are also visible outside of the skin surface, as can be seen in Fig. 2a, and are therefore included in the reconstruction, as seen in Fig. 2b. Unfortunately, such artifacts are very hard to eliminate in post-processing of imaging data, and therefore, these artifacts interfere with the reconstruction of the patient’s skin surface. In fact, the true surface structure of the patients’ skin cannot be recovered from the affected CT images, since the artifacts occur directly when the image is initially generated. Figure 2b also shows that since grey values within the volume are not homogeneous, the 3D objects are not closed, which would make 3D printing complicated and expensive, if even possible. To create 3D models suitable for low-cost 3D printing, the STL files exported from InVesalius were post-processed using meshmixer (http://www.meshmixer.com/), a free software which is tailored to the generation of models for 3D printing. As a first step, the region of interest (i.e. the head of the patient) was cropped from the total volume using meshmixer’s “plane cut” functionality. Cropping was performed in a manner to result in two plane surfaces - this allows the stable placement of printed models on a plane surface in either horizontal, supine or vertical position. The remaining volume was closed, resulting in a watertight mesh, using the “close cracks” and “make solid” functions in meshmixer. “Make solid” also allows for smoothing of the model, by adjusting the accuracy and density of the resulting closed mesh, and by specifying the desired minimal thickness of the model, which was chosen as five mm. Any remaining artifacts, in example from metallic dental material, were manually removed using the “Sculpt” tool. Additional smoothing was manually performed, if necessary, by selecting noisy surfaces and applying the “Deform - Smooth” function. It is important to point out that the manual dental artifact reduction can, in some cases, lead to some loss of detail in the area of the mouth and lips in our 3D models. Furthermore, the transformation of the mesh within the world coordinate system was adjusted to make the model fit as a whole within most common 3D printers. As a last step, a cylindrical volume was cropped from the back of the head, reducing the total volume of the model and therefore, reducing printing material and printing cost. This step is performed by adding a cylindrical mesh to the scene, fitting it to the model and computing the “Boolean Difference” between the meshes. An example of the final 3D model is shown in Fig. 2c). Finally, to make our 3D models usable with a large variety of visualization software, we additionally exported our models as Wavefront OBJ (Wavefront, Santa Barbara, CA, USA) files. The final STL files, optimized for 3D printing, and OBJ files are available on figshare^16^.

## Data Records

The final data collection has been stored in a figshare repository^16^. The anonymized PET/CT scan and 3D facial model collection is stored as single NRRD and STL files, which are organized by anonymized patient ID numbers (’patID’, e.g., “pat1_CT.nrrd”, “pat1_ PET.nrrd”, “pat1.stl”) and can be cross-referenced against data Tables 1 and 2 using this identifier.

**Table 2:** Patient metadata for cases included in the study. Gender categories M for male and F for female are used. The tumor entity was an oral squamous cell carcinoma (SCC) in all cases. The TNM Classification of Malignant Tumors (TNM) is reported to categorize the primary tumor site (T), the regional lymph node involvement (N) and the presence of distant metastatic spread (M).

Patient	Age	Sex	Body Weight	Body Height	Pathology		
	(years)	(F/M)	(kg)	(cm)	Tumor Entity	Location	TNM
1	45	M	70	182	Oral SCC	Floor of Mouth, Central Mandible	T4N1M1
2	64	M	110	162	Oral SCC	Left Mandible, Neck	T0N3M0
3	72	M	65	166	Oral SCC	Right Tongue	T1N0M0
4	58	M	55	168	Oral SCC	Floor of Mouth, Left Mandible	T2N2M0
5	67	F	58	159	Oral SCC	Left Mandible	T4N0M0
6	56	M	60	178	Oral SCC	Right Mandible	T4N0M1
7	50	F	67	167	Oral SCC	Floor of Mouth, Left Mandible	T2N2M0
8	61	M	73	127	Oral SCC	Floor of Mouth, Right Mandible	T2N1M0
9	57	M	58	177	Oral SCC	Right Retromolar Triangle	T4N0M0
10	65	F	81	178	Oral SCC	Floor of Mouth, Right Mandible	T2N0M0
11	70	M	64	180	Oral SCC	Left Floor of Mouth	T4N0M0
12	81	M	80	172	Oral SCC	Right Cheek	T1N0M0

The volumetric image data stored in NRRD file format includes complete physiological faces and the patient-specific anatomical facial structures. The 3D models of the facial surface are provided within this data collection as printable STL files. For a visual de-identification in the reconstructed 3D data sets, all eyes were closed during the data record procedure, similar to 2D photos. These data sets, resulting from the presented data selection and processing steps, are described in Table 1. For PET/CT data sets, voxel measurements and resolution (in-plane resolution and slice thickness) are listed. For 3D models, we report the total volume sizes, as they are important information for 3D printing. Knowing the total volume sizes, a suitable 3D printer model can be chosen, and total cost can easily be estimated.

The collection consists of 12 anonymized PET/CT scans of the human cranio-maxillofacial complex in NRRD format, which originate from full body volumetric PET/CT scans in DICOM format retrospectively taken from the clinical routine in head and neck oncology (Department of Oral and Maxillofacial Surgery, Medical University of Graz, Austria). The collected cases for the study are described in Table 2. For each patient, the age, sex, body weight, body height as well as the type and anatomical location of the pathology including the according TNM Classification of Malignant Tumors (TNM) for squamous cell carcinomas (UICC 2017) are noted. TNM describes the size of the primary tumor and wether it has invaded nearby tissue (**T**), nearby involved lymph nodes (**N**) and distant metastasis (**M**)^20^.

One limiting factor of the presented data collection is its relatively small size. A higher amount of cases would allow our data collection to be translated to other research areas, like simulation or deep learning, more easily. However, combined clinical PET/CT scans are usually not widely available, even in big clinical centers, since these investigations are highly cost intensive and need both a large amount of radiological equipment and human resources. Therefore, routinely performed PET/CT scans are rare, even in big head and neck cancer centers. Furthermore, as highlighted in this investigation, our strict inclusion criteria forced us to exclude many of the available cases, due to limited imaging quality, misalignment between PET and CT scans, or strong artifacts in the region of interest. Another aspect we would like to point out is that informed consent had to be obtained from all selected patients prior to publication of their data. Our data collection contains identifiable patient data, not only in the form of medical imaging, but also as 3D models of the face, so obviously, obtaining informed consent of patients for publication of this data is limiting factor.

## Technical Validation

The main issue regarding technical validation of the data set is the spatial geometry of the medical imagery, as reconstructed by the CT or PET/CT scanner software. Medical CT or PET/CT scanners used for this data collection are regularly subjected to quality control evaluations and are certified medical products^21^, since they are routinely used in daily clinical practice. PET/CT scanners are under the responsibility of a qualified medical staff and medical physicists^22,23^.

To validate the generated 3D models of the patients facial structure (STL files), the models were 3D printed using a commercial 3D printer, the Ultimaker 2 Extended+ (Ultimaker, Utrecht, Netherlands). This printer is also used in clinical head and neck reconstruction centers for the creation of patient-specific 3D template models pre-operatively. Printing was performed using a layer resolution of 0.1 mm, a filling level of 10% and polylactide as a material, resulting in a cost of approximately 30 EUR (34 USD) per model, making them a very low-cost alternative to commercially available patient phantoms or dummy heads. An example of such a print is shown in Fig. 2d. The patient phantoms were subsequently successfully used for the evaluation of an image-to-face registration system for head and neck surgery using a commercially available AR headset, the Microsoft HoloLens (Microsoft Corporation, Redmond, WA, USA)^24^.

## Usage Notes

For research purposes, the data can freely be downloaded, but we kindly ask investigators to cite our work. The data provided within this work is free to share. The material can be copied and redistributed in any medium or format. Furthermore, the data is free to adapt - remix, transform, and build upon the material. The data within this work is licensed under a Creative Commons Attribution 4.0 International License (CC BY 4.0) (https://creativecommons.org/licenses/by/4.0/).

### Medical image data processing

NRRD files can be processed with free or open source medical imaging platforms, such as MeVisLab (https://www.mevislab.de)^25^, 3D Slicer (https://www.slicer.org)^26^ or Studierfenster (http://studierfenster.at)^27^, and are supported by programming languages such as MATLAB (https://www.mathworks.com) or Python (https://www.python.org/) using publically available modules or libraries. Furthermore, a wide variety of commercially available image processing tools are available for advanced image segmentation (e.g. for the segmentation of the skin surface from CT or the segmentation of other structures of interest) and analysis, such as Mimics (Materialise NV, Leuven, Belgium, https://www.materialise.com/medical/software/mimics) or Simpleware (Synopsis, Mountain View, CA, USA, https://www.synopsys.com/simpleware.html).

### 3D visualization and processing

STL files are widely used for rapid prototyping, 3D printing and computer-aided design and manufacturing^28^. They can be analyzed, manipulated and processed with open source and free software tools such as Blender (https://www.blender.org/), MeshLab (https://www.meshlab.net), meshmixer (http://www.meshmixer.com/). Commercially available tools for STL manipulation include Photoshop (Adobe Inc., Mountain View, CA, USA, https://www.adobe.com/products/photoshop.html), and software specifically designed for additive manufacturing, design and professional 3D printing, such as Netfabb (Autodesk, San Rafael, CA, USA, https://www.autodesk.com/products/netfabb) or Magics 3D Print Suite (Materialise NV, Leuven, Belgium, https://www.materialise.com/en/software/magics-3d-print-suite). Certainly, since STL files are supported by most software packages coming with commercial 3D printers, the STL files provided within this data collection can directly be printed without further manipulation.

## Data Availability

Aside from the image and 3D model processing steps performed with free or open source software described in this section, we used a custom script written in Python 3.6 with NumPy 1.15.4 (https://numpy.org/) for cropping CT and PET scans in NRRD format, to cover the head and neck region only. This script furthermore outputs information about the volumetric medical data, such as the size and resolution, as seen in Table 1. This python script utilizes the pynrrd 0.4.0 library (https://pypi.org/project/pynrrd/) to parse information about slice resolution and volume size from the header of NRRD files. With this information, we calculate the ideal number of slices to be included in the final NRRD files for upload, as described by formula. These slices are then again automatically converted to NRRD format and saved to disk. These are the NRRD files available in our figshare repository^16^. The Python script is available on GitHub (https://github.com/cgsaxner/PET-CT_preprocessing).
